# Psychotherapists’ perspectives on collaboration and stepped care in outpatient psychotherapy—A qualitative study

**DOI:** 10.1371/journal.pone.0228748

**Published:** 2020-02-05

**Authors:** Kerstin Maehder, Bernd Löwe, Martin Härter, Daniela Heddaeus, Olaf von dem Knesebeck, Angelika Weigel

**Affiliations:** 1 Department of Psychosomatic Medicine and Psychotherapy, University Medical Center Hamburg-Eppendorf, Hamburg, Germany; 2 Department of Medical Psychology, University Medical Center Hamburg-Eppendorf, Hamburg, Germany; 3 Department of Medical Sociology, University Medical Center Hamburg-Eppendorf, Hamburg, Germany; Universitetet i Stavanger, NORWAY

## Abstract

**Objective:**

Stepped and collaborative care with outpatient psychotherapy as one treatment step is guideline-recommended for mental health care. To date, the experiences and evaluation of psychotherapists regarding collaboration and stepped care have been neglected. In order to improve collaborative mental health care, this qualitative study aimed at identifying psychotherapists’ perspectives and needs within collaboration and stepped care.

**Methods:**

Semi-structured qualitative interviews with 20 German outpatient psychotherapists were conducted and analyzed applying thematic analysis. The analysis was realized in a recursive process to first identify themes and then relate these themes back to the research questions with regard to collaboration and stepped care.

**Results:**

Collaboration mainly took place in small networks, with general practitioners and psychiatrists as the most important partners and psychotherapists wishing to intensify collaboration. Main barriers for collaboration were seen in deficient resources and remuneration and in a perceived lack of esteem by other medical specialties. Stepped care was appreciated for intensified collaboration and low-threshold access to care. Doubts were cast on its implementation within current health care conditions, worries concerned a primacy of economic principles instead of patient-orientation. Among further needs, psychotherapists demanded increased knowledge about psychotherapy, especially among general practitioners.

**Conclusion:**

Psychotherapists expressed ambivalent attitudes towards stepped and collaborative care, substantially influenced by health care conditions and the perceived own standing among care providers. Psychotherapists’ needs within stepped care comprise intensified collaboration, sufficient time, personal and financial resources for collaboration and opportunities for a constructive interprofessional dialogue.

## Introduction

Despite an open access mental health care system in Germany, patients with mental disorders are often underdiagnosed and underserved [1]. Stigmatization and a lack of resources in specialized health care contribute to the problem and the fragmentation of health care in different settings further impedes diagnostics and treatment [2, 3]. This fragmentation includes the insufficient collaboration between primary care and specialized mental health care since most patients access health care via general practice [4, 5]. To improve health care for patients suffering from mental disorders, German and international guidelines recommend collaborative care as an evidence-based care model [6–8]. Additionally, to account for both limited health care resources and patient needs, it is advised to organize care in a stepped care model [6, 7]. Stepped care is defined by two core features: 1) a care algorithm with treatment steps increasing in intensity, allocated according to disorder severity and 2) systematic monitoring to regularly assess whether the treatment needs adaptation [9]. Stepped care models for depression, anxiety or somatoform disorders have proven to be mostly effective and cost-effective. However, further research is needed to clarify facilitators of a successful implementation in different health care settings and for a broader range of disorders and comorbidities [10–15].

The transition of guideline-recommended collaborative and stepped care into routine care in order to improve care for patients with mental disorders depends on the acceptability among all involved care providers. Thus, a first step with regard to implementability is to take stock of the care providers’ perspectives on both collaboration experiences and the concept of stepped care. However, to date, stepped care has been evaluated from the viewpoint of e.g. general practitioners (GPs) and patients [16–18]. Despite a generally high acceptance of the concept among providers and patients [16], previous implementations were often hindered by structural barriers in everyday practice (e.g. lack of time) and depended on care provider competences and roles (e.g. mental health nurses) and motivation [19]. Skepticism was expressed towards pre-determined treatment steps, especially the low-intensity treatment options [18, 19], and partly towards monitoring when based on questionnaires instead of clinical evaluation [19]. GPs welcomed the idea of stepped care especially for improved collaboration [17]. For patients, the access to a stepped care pathway via their GP seems to be an acceptable and helpful way to mental health care [19]. In an earlier German cross-sectional questionnaire study, stepped care elements, for example the intensified collaboration and systematic monitoring, were implemented and mainly evaluated positively by the participating GPs, psychotherapists and psychiatrists [20]. Yet, in the evaluation of the general concept of stepped care, outpatient psychotherapists’ perspectives and needs, have mostly been neglected, despite psychotherapy being an integral part of guideline-recommendations and health care services for mental health problems. As psychotherapists in Germany do not yet work as part of structured stepped care models, the current collaboration experiences of psychotherapists have to be considered to inform future implementation of stepped care and to make use of the valuable contributions this care provider group can add.

Thus, the aims of the present qualitative study were to gain insights into the perspectives of outpatient psychotherapists in Germany regarding their current experiences with interprofessional collaboration and their evaluation of stepped care. The specific research questions were: *How do outpatient psychotherapists in Germany experience and evaluate collaboration with other care providers*? *How do outpatient psychotherapists evaluate the concept of stepped care in general and with regard to collaboration*? Based on these insights, psychotherapists’ needs for improving collaboration and for implementing stepped care will be deduced and discussed, with the ultimate goal of ameliorating patient care.

## Methods

### Qualitative approach

To gain a deeper understanding of the psychotherapists’ perspectives, a qualitative approach was chosen, as this allows to explore the psychotherapists’ experiences and the way they attach and express meaning to respectively on these experiences with regard to collaboration and stepped care. Yet, to depict relevant aspects within these topics, the analysis was planned as rather descriptive instead of interpretative. The study was conducted within the qualitative framework of thematic analysis, as a flexible yet structured approach to engage with qualitative data [21]. As Braun and Clarke point out in their seminal paper on thematic analysis, this qualitative approach can be used within a variety of theoretical and epistemological stances [21], but the choice of epistemological and ontological paradigms a study is based on should be made transparent. As the research questions of this study focused on experiences and evaluations of psychotherapists (so assuming a shared reality with e.g. collaborators) instead of on the structural and socio-cultural conditions that shape the psychotherapists’ sense-making of their profession (which would require a constructionist perspective) the following decisions were made: As ontological stance, a critical realist standpoint was adopted (set between “realism” and “relativism”, [22]), which assumes that reality is always socially influenced and only partially accessible but that there is enough concordance in reality perception to gain meaningful insights [22]. As to the epistemological paradigm, the study was realized within the position of “contextualism” (set between “positivism” and “constructionism” [22]), supposing that there is no single reality that can be grasped but that reality and its perception are always bound by context and perspective. Bearing this in mind, data can be considered an informative source for experiences and individual or collective sense-making. Hence, while the psychotherapists’ perspectives cannot be assumed to be “true”, their views influence the health care provision for patients and, thus, have to be taken into account with regard to collaboration and stepped care. While the analysis of the data inevitably is primed by the researchers’ subjectivity, the intersubjective interpretation of the data within its context and within the context of research literature allows for insights that may improve patient care through better collaboration and a needs-based and resource-efficient mental health care.

### Set-up of the study

The present study was conducted in the context of a randomized-controlled trial on stepped care for patients with depression, anxiety, somatoform and alcohol-related disorders (COMET, Clinical trial registration No. NCT03226743; [23]). Psychotherapists were recruited inside and outside the RCT-cohort. However, the RCT had not started to recruit patients at the time of data acquisition which prevented interviewees from the COMET-group from prior experiences with stepped care.

### Participants

Theoretical sampling was chosen, aiming at a diverse sample of participants with differing characteristics regarding: gender, age, years of professional experience, psychological vs. medical background, psychotherapeutic approach (one of the approaches reimbursed by health insurance in Germany: psychodynamic, psychoanalytic or cognitive-behavioral therapy [24]) and (non-) participation in the COMET-trial. These criteria were chosen because of their possible influence on collaboration experiences and expectations within the health care system, as e.g. a longer time in practice allows for more network building and the different educational paths come along with different professional socialization and affiliations.

During the recruitment phase between April and July 2018, registered outpatient psychotherapists in the metropolitan area of Hamburg in Germany were approached via phone or email. For the psychotherapists not taking part in the RCT-trial, the register of the local Association of Statutory Health Insurance Physicians was used to randomly contact psychotherapists according to the sampling criteria. Recruitment of participants was stopped when further interviews did not provide relevant new insights, thus an acceptable level of data saturation was reached as consented in discussion by AW, KM and MW.

### Data collection and processing

Based on the current literature on collaborative and stepped care, a semi-structured interview guide was developed to initiate interviewees’ reflections on the questions of collaboration and stepped care, while allowing for further inquiry on emerging topics (S1 File). Interviews were conducted either via phone or face-to-face according to interviewees’ preferences. Since the concept of stepped care was still widely unknown, a short standardized description of the basic principles was presented. Interviewers were female, with a background in psychology (KM, MW). Interviews were audiotaped, transcribed verbatim (while assuring pseudonymization) and analyzed using the software MAXQDA (MAXQDA Standard, Release 18.2.0, VERBI GmbH, 2018).

### Data analysis

Qualitative data analysis followed the suggested steps for thematic analysis [21]. At first, familiarization with data was reached by transcription of the audio files, by reading the transcripts and reflecting on the first impressions. Next, initial codes were independently generated for all transcripts. Themes were then inductively developed by condensing codes to meaningful units, supported by discussing code mind maps. These themes were reviewed by relating back to the original material. In order to answer the research questions the focus was then set on the two main topics of collaboration and stepped care, to allow insights into these specific aspects. Thus, a deductive approach was followed to identify codes and themes in relation to the research questions and the interview guide. Accordance and discord between the interviews were reflected on as were the researchers’ pre-assumptions and interpretations. In order to delineate themes from each other, these were defined and labeled and illustrative quotes were identified. In a last step, central themes were structured and reported to answer the research questions regarding the topics of collaboration and stepped care. Within the topic of collaboration, a focus was set on the two other central professional groups in outpatient mental health care (GPs and psychiatrists). Techniques to enhance trustworthiness included co-coding 14 out of 20 transcripts and the discussion of results within the current research literature. Additionally, a one-hour workshop with registered psychotherapists (other than those interviewed within the study) and health services researchers was conducted in order to realize a member-check of the results. Member-checking largely supported the identified themes, especially the barriers to collaboration due to the health care system, but pointed out to the limited self-reflectiveness of the interviewees with regard to barriers on the psychotherapists’ side. Reporting of the results followed the Consolidated Criteria for Reporting Qualitative Research (COREQ)-guideline [25]. The clear regional boundaries (i.e. the city of Hamburg), the qualitative approach and the recruitment of participants within a still ongoing RCT bear the risk of identification of study participants in case of a provision of individual data. Thus, in accordance with the informed consent and the data protection regulations, data can only be made available upon request to the authors. However, to allow for further insights, the MAXQDA codebook can be found in S2 File.

### Research ethics

The study complies with the Declaration of Helsinki [26]. The COMET-trial, in which context the current study was conducted, was approved by the ethical committee of the chamber of physicians in Hamburg (No. PV5595). All participants provided written informed consent. Data protection was in accordance with the European General Data Protection Regulation, as implemented on May 25^th^ 2018 [27].

## Results

### Participants

According to the sampling criteria, 46 psychotherapists were contacted of whom 19 did not respond and seven refused to participate due to time pressure, while 20 agreed to participate and were interviewed (70% female). The age of interviewees ranged between 34 and 64 years (M = 52.9 years, SD = 9.6) and years of experience between 5 and 33 years (M = 18.9 years, SD = 9.9). Based on the educational background in psychology (55%) or medicine (45%), 55% were trained in psychodynamic therapy (with three participants additionally trained in psychoanalytic therapy) vs. 45% in cognitive-behavioral therapy. Interviews had a mean duration of 33 minutes (range 22–53 minutes, SD = 7.2).

### Thematic analysis of the psychotherapists’ perspectives on collaboration and stepped care

Results on psychotherapists’ current collaboration experiences (1) will be presented, followed by their view on stepped care (2). Quotes were edited for legibility and explanations have been added in square brackets where necessary. Quotes are identified by medical (M) vs. psychological (P) educational background and the psychotherapeutic approach of either psychodynamic (PD) or cognitive-behavioral therapy (CBT).

#### (1) Psychotherapists’ perspectives on current collaboration

After an overview on the psychotherapists’ general collaboration experiences, more detailed accounts on the collaboration with the most important collaboration partners and key actors in mental health care, i.e. a) GPs and b) psychiatrists will be given. Moreover, themes that were identified as c) overarching interprofessional challenges between psychotherapists and other medical specialties will be presented.

As a first overview on collaboration, psychotherapists identified GPs as most important collaboration partners, followed by outpatient psychiatrists. Among further collaborators of less overall importance were inpatient and day clinics, medical specialties such as neurology, counselling services and self-help groups. The overall intensity of collaboration varied from rarely to regularly, with most psychotherapists wishing for more collaboration. Yet, not all interviewees saw a closer collaboration as necessary: “*I’d say*, *each of us is doing his job*.” (M, PD). Higher intensity of collaboration was often linked to small local networks built over time with GPs and/or psychiatrists with whom the interviewees “*felt to be on the same wavelength*” (M, PD).

Collaboration was only initiated or intensified when needed, due to omnipresent time pressure and unavailability by phone on both the psychotherapists’ and the collaborators’ side. While the interviewees mainly considered themselves as initiators of collaborative exchange, some critically reflected on their own lack of initiative to foster collaboration and to improve availability for collaborators. The structural barriers of time pressure and unavailability played a major role in the accounts of the psychotherapists and were further reinforced by the lack of remuneration for collaborative activities between these professional groups in German health care. Reasons for collaboration were somatic differential diagnosis and somatic health care, formal constraints (such as a short medical report necessary for the reimbursement of psychotherapy), complex or acute cases or the transition between general and mental health care. The collaboration with inpatient and day clinics was considered to be of comparably lower importance to psychotherapists’ daily practice. However, this collaboration was equally impeded by the barriers of time pressure and mutual availability and facilitated by personal contacts.

Regarding the collaboration with GPs, psychotherapists reported heterogeneous experiences. Apart from relevant structural barriers (e.g. time pressure), the GPs’ varying interest and competency in mental health care were considered as main influence on collaboration. GPs were generally assumed to be rather open-minded towards mental health problems compared to other medical specialties.

The main benefit of collaborating with GPs was seen in exchanging valuable information on the patient from both perspectives: While GPs often had long-term relationships with their patients and a practice team adding further information, psychotherapists pointed to the depth of their psychotherapeutic work. With regard to referrals, psychotherapists valued some GPs’ good intuition for a patient’s openness for psychotherapy or their preparatory work: “*Sometimes*, *GPs had already invested months or even years to persuade the patients so that they are willing to say ‘Okay*, *I’ll give this shrink a chance*” (M, PD).

In contrast, other GPs were deemed to not assume mental health as part of their responsibility, only dealing with their patients’ mental health concerns because of the shortage of psychiatrists. In some cases, the contact with GPs was described as *“strenuous”* (P, CBT), leaving the notion among the interviewees of not being valued or treated as equal and important care providers: *“I don’t think that they really see a sense in what we’re doing*.*”* (P, CBT).

One major and recurring criticism issued by psychotherapists was the lack of mental health competencies in GPs: *“[…] But there are many*, *many other [GPs] (…) who go through their whole array of somatic diagnostics and then end like ‘could be something mental’*.*”* (M, PD)

This resulted in varying degrees of frustration or even anger among the interviewees: *“They’re often not trained well and yet they think they are*. *This is when it gets difficult*.*”* (P, CBT). Specifically, interviewees criticized GPs for sometimes being too quick in labelling patients as mentally ill, for prescribing psychopharmacotherapy too often, for putting patients on sick leave too early or too long or for referring patients without appropriate indication for psychotherapy.

The collaboration with psychiatrists was strongly influenced by the perceived shortage of outpatient psychiatrists, resulting in an overload of available practices:

*I’d like to have someone [a psychiatrist] saying ‘If you call me and want to refer your patient to me*, *you’ll get an appointment within a week’*. *I really do find it hard*, *if I say ‘I do have a suicidal patient sitting here*, *could you please take a look at him today*?*’ and the answer is ‘Sorry*, *I’m full’*. (M, PD)

The collaboration intensity with psychiatrists varied, with only a few interviewees being satisfied and most wishing for more exchange. As with GPs, collaboration was facilitated within small networks, while contact with unknown psychiatrists or neurologists was seen as *“practically non-existent”* (M, PD).

Disregarding these difficulties, interviewees described the exchange as mostly cooperative, especially when based on previous personal encounters. The psychiatrists’ professional evaluation on specific patients was partly valued as feedback for the psychotherapists’ own impression. Asked about what psychiatrists might think about psychotherapists, one interviewee suspected that *“They [the psychiatrists] laugh about us*. *Because*, *I think*, *we don’t present ourselves as one coherent group and we don’t act as competent in every aspect as we could*.*”* (P, CBT).

As part of the psychotherapists’ perspectives on collaboration, overarching interprofessional challenges between psychotherapists and other medical specialties became apparent.

A major hindrance to collaboration was seen in general “*territorial claims*” (P, PD) between the different specialties, partly due to questions of power but also due to different professional socializations. For instance, psychotherapists criticized that sometimes other medical specialties treated patients‘ symptoms from an exclusively biomedical perspective:

*Sometimes patients tell me that their orthopedist treats their pain with injections that only help for a few days or so*, *without treating the underlying problem*. *Or a patient gets x-rayed or has an MRI (…) without ever looking beyond*. (P, CBT)

While one interviewee admitted that every specialty had its own *“codes”* and *“models”* (M, PD), a too rigid biomedical focus was considered as hindrance to a constructive interprofessional dialogue and as barrier to a patient’s willingness to engage in psychotherapy: *“I experience some physicians as narrow-minded*, *those who fix patients on a specific model in a problematic way*. *Yet*, *in everyday practice there is no constructive dialogue on this*.*” (M*, *PD)*. Moreover, some psychotherapists criticized that physicians were often ill-informed about psychotherapy and the different psychotherapeutic approaches: *“They have little insight into what psychotherapeutic work actually is and how diverse psychotherapeutic work can be*.*”* (P, CBT).

While interviewees deplored the perceived lack of esteem for psychotherapy by some medical specialties, this impression was further reinforced by the general feeling of devaluation that some psychotherapists experienced within the health care system, e.g. by an insufficient remuneration or additional regulations:

*You don’t gain this impression [that psychotherapy is valued] in our health care system (…) although everyone cries for it and everyone says ‘Mental disorders are on the rise’*. *You don’t get the impression that hindrances are removed*. *And it really would help if things got easier instead of new regulations all the time*. (M, PD).

#### (2) Psychotherapists‘ perspectives on stepped care

In the following, psychotherapists’ perceived benefits as well as barriers and disadvantages on stepped care will be presented with regard to (a) the concept in general and (b) with regard to collaboration. As described, the evaluation mainly relied on the standardized input on stepped care, not on previous practical experiences.

From the psychotherapists’ perspective, the general concept of stepped care was partly appreciated for its benefits. Stepped care was considered a chance for timely recognition and treatment of mental disorders, for “*using the possibilities to turn around early when symptoms arise*” (P, CBT). The diversification of care in different steps was generally welcomed as useful for the allocation of resources and for low-threshold services for patients still reluctant to engage in psychotherapy. Seen from the patients’ perspective, psychotherapists partly appreciated that GPs should function as access to stepped care: *“If a GP is involved*, *this would probably make it easier for a patient to understand himself within the health care system*.*”* (P, CBT). Structured care pathways could allow for a better overview of the available treatments for both care providers and patients. To foster this, one psychotherapist suggested to establish *“a network coordinator”* (P, CBT).

With regard to monitoring, as second core principle of stepped care, the interviewees saw an opportunity to regularly review the therapeutic progress and to allocate resources according to needs, rather than only exhausting the predefined contingent of psychotherapy sessions as granted by the insurance.

In contrast to the benefits, psychotherapists also identified barriers and disadvantages in the concept of stepped care. While the stepping of care was generally considered as useful, several psychotherapists questioned the novelty of the approach: *“Don’t we do this already*?*”* (P, CBT). Regarding the conception of treatment steps and care decisions for individual patients, the interviewees worried that their proficiency would not be adequately considered, e.g. that they would not be allowed to self-dependently adapt the number and frequency of sessions to a patient’s need.

Concerns were raised that within stepped care the treatment might no longer follow the *“organic”* (M, PD) psychotherapeutic process that allows for symptom fluctuation as part of progress without being immediately tied to stepping-up/down-criteria. This concern corresponded to the objections against monitoring since psychotherapists (especially with psychodynamic background) questioned the measurability of therapeutic progress with questionnaires: *“Depending on the state a patient is in and whether he currently is in a conflict with his therapist*, *the results might be really weird*.*”* (P, PD).

In addition, regular measurements would increase the administrative burden which generally was a predominant worry raised against stepped care. Major opposition was pronounced against the idea that stepped care could primarily serve economic principles like cost-effectiveness and cost-savings at the expense of treatment intensity and length a patient might need: *“The patient might get the feeling of being under pressure because he is constantly asked how he feels and when he’ll finally function again*.*”* (M, PD). For the same reason psychotherapists feared the potential replacement of psychotherapy by unguided e-health interventions within stepped care, both with regard to patients with mild disorders who might be *“put off with some kind of computer program”* (M, PD) and with regard to the mental hygiene of psychotherapists *“because it’s good if*, *on the one hand*, *I treat severely ill borderline patients (…) and*, *on the other hand*, *a first depressive episode where treatment is going smoothly*.*”* (P, CBT).

Additionally, the interviewees argued for retaining patients’ free choice of care providers and for not predetermining care pathways too tightly as this could *“nip the patients’ own responsibility in the bud”* (M, PD) despite this being considered as important for the therapeutic process.

As to the benefits of stepped care for collaboration, the intensified collaboration within stepped care was considered the most important benefitespecially between GPs and psychotherapists, provided more time and remuneration for collaboration was available. More collaboration was deemed particularly useful regarding complex cases and cases with relevant somatic and mental symptoms, such as somatoform disorders or comorbidity of chronic health conditions and mental disorders.

Several barriers and disadvantages of stepped care for collaboration were described by the psychotherapist. Although the interviewees unanimously welcomed the idea of fostered collaboration, its implementability within the present health care system was challenged. Besides the lack of time, resources and remuneration, one recurring criticism addressed the deficient mental health competencies of GPs, who would play a major role within a stepped care model anchored in primary care. Thus, further GP training and earlier requests of psychotherapeutic competencies in the diagnostics and treatment process were suggested as precondition for successful stepped care. These reservations against decision power transferred to less competent care providers were fostered by the aforementioned impression of some interviewees that psychotherapists were not adequately valued.

Thus, while stepped care was overall deemed a positive concept, especially with regard to collaboration, skepticism concerning its implementation and the appropriate consideration of psychotherapists within it was distinct.

## Discussion

The current qualitative study shed light on how outpatient psychotherapists in a German metropolitan area experience collaboration with other care providers and how they evaluate the concept of stepped care, both generally and with regard to collaboration.

To summarize, most interviewees have arranged for collaboration in small local networks with GPs and psychiatrists but wish for more collaboration. Main barriers were seen in time pressure, the lack of health care resources and the deficient remuneration of collaborative activities. Throughout the accounts, interprofessional discord with other medical specialties emerged, both with regard to power and esteem within the health care system and with regard to the treatment of individual patients.

The concept of stepped care was generally welcomed but doubts were cast on its implementation within the current health care conditions, e.g. time, financial and personal resources. Psychotherapists suspected a shift of decision power to instances with too little mental health competencies. Worries emerged that stepped care might primarily be oriented towards economic principles.

The identified main barrier for collaboration, the lack of time, resources and remuneration is an experience shared by many care providers [28–31]. To foster collaboration, these structural conditions have to be improved, e.g. enough time for personal encounters between health care providers that the interviewees considered as crucial to create networks. Hermens et al. [17] equally underline the importance of stakeholder support, e.g. through politically established financial incentives for collaboration. If stepped care, as guideline-recommended model of care [6, 7] is adopted, it cannot resolve the overall deficiency in resources within the health care system but may add to a more efficient allocation of resources via structured care pathways and better linkages between the different actors involved [32–34].

In addition to these pivotal barriers, the following needs of psychotherapists with regard to stepped care models were identified. If stepped care is to be both acceptable and implementable, these identified needs should be considered as practice implications

The general idea of stepped care was evaluated positively by psychotherapists in this study, especially for the intensified collaboration, in congruence to GPs’ and patients’ evaluation in other studies [16, 17]. As to the design of stepped care pathways, psychotherapists refer to their mental health competencies when arguing for an earlier involvement in diagnostics and treatment, as suggested in guidelines [7]. Self-help options as a low-intensity treatment within stepped care were seen critically, in line with the GPs’ opinion in the study of Franx et al [18] and the limited use of these treatment options reported in Hermens et al. [17]. In the patient and provider survey by Haugh et al. [16], about half the patients found low-intensity options to be acceptable, at a same level with medication, while psychotherapy met with 72% approval. While the low-threshold access to stepped care via general practice was welcomed, as it is the most common point of access to care at present [4], further training of GPs in mental health and an increased knowledge about psychotherapeutic work and, psychotherapeutic approaches for all medical specialties concerned was deemed necessary. The lack of knowledge has already been identified as a barrier for collaboration [35] and closing this gap might help e.g. GPs to better prepare patients prior to a referral to psychotherapy. However, this equally calls for the psychotherapists to increase their knowledge on other medical disciplines and to become more transparent and communicative about their own work. From the GPs point of view, the lack of communication equally is a lack of information flow from the specialized mental health sector to general practice, as expressed in the study on stepped care implementation by Hermens et al. [17]. By fostering both collaboration and knowledge on treatment options for *all* care providers and patients, stepped care could counteract care fragmentation [2, 3] and improve mutual understanding, as it has been realized in multi-professional teams in a stepped care approach for depression [18].

Based on the study results, further features could render a stepped care model more acceptable to psychotherapists. Stepped care should not significantly increase the already high administrative burden [36, 37]. Yet, the administration of systematic monitoring instruments, as a central feature of stepped care, has been considered time-consuming in the study of Franx et al. [18]. However, in contrast to the rather critical stance of the interviewees in this study, GPs and patients still found the monitoring to be helpful for both treatment decisions and course evaluation [18]. While structuring care was considered useful, stepped care should remain patient-oriented and allow for greater flexibility with regard to number and frequency of therapy sessions, neither primarily serving economic aims nor suffocating patients’ own initiative.

Besides these concrete aspects to increase acceptability of stepped care for outpatient psychotherapists, the present study revealed how much questions of professional standing and decision power played a role in collaboration experiences and the evaluation of stepped care. Thus, when building care networks within stepped care, attention should be payed to adequately map and value each care providers’ competencies, including the psychotherapists, underlining the mutual additional value rather than the competition.

Although this study has been conducted within the German health care system, the identified needs of psychotherapists with regard to collaboration and stepped care echo findings from studies set in other countries [28–31, 35, 37]. Yet, when conceiving and implementing stepped care within different health care systems, additional facilitators and barriers should be considered, e.g. the opportunities of co-location of GPs and mental health specialists as fostered in the UK initiative Improved Access to Psychological Therapy (IAPT) [38], the different models of access to and reimbursement of psychotherapy [39], the organization of primary care in interprofessional clinics [40] or the opportunities for collaboration that arise with shared electronic records and secure communication channels [41]. Besides these structural aspects, the present study with its qualitative design revealed the major importance of interprofessional tensions and fears as highly relevant factor with regard to the successful implementation of stepped and collaborative care models.

### Strengths and limitations

While the overall consistency of themes was rather high, the qualitative approach and the small sample size prevent from uncritically generalizing the results. Yet, the results match current research literature and the diversity regarding the sampling criteria facilitated a broad spectrum of possibly differing experiences. However, interviewees were all part of the same professional group so that the perspective on collaboration inevitably covers only their side and their profession’s interests. Furthermore, the interviewees focused on external barriers and deficits (e.g. within the health care system or in other care providers), while reflections on their own potential limitations regarding collaboration were marginal. As the study was set in a metropolitan area in Germany, collaboration experiences were clearly influenced by both the German health care system and the metropolitan character with its higher density of psychotherapists and other care providers. Still, most topics, such as time pressure, interprofessional challenges and the fear of too much economic dictation have been reported from other countries [28–31, 35, 37] and, thus, seem to be of great importance beyond the German health care system. Further research is, thus, needed with regard to other professions’ and patients’ perspectives on stepped care, with regard to rural areas and with regard to the implementability within the current health care system and the necessary changes, e.g. remunerating collaborative activities. As collaboration is central to optimal mental health care, ways to better connect the respective professional groups and to foster exchange should be promoted and evaluated, for example models of co-location such as within the UK initiative IAPT [38] or tele-health options, where psychotherapists can offer diagnostics and treatment in GP practices [42]. Additionally, the qualitative approach should be complemented by quantitative research to allow for generalizability and statistical evaluation.

## Conclusion

Currently, psychotherapists’ perspectives on collaboration in a German metropolitan area reveal limited collaboration in small local networks and the wish for intensified interprofessional exchange. The main barriers were identified in the current structures of the health care system, especially with regard to time, resources and remuneration. While stepped care was considered a useful approach that might facilitate early recognition and foster collaboration, its success and acceptability depend on a change in the current health care structures, the consideration of psychotherapists’ needs within stepped care models and on a constructive dialogue between the different professional groups involved. These aspects are taken into consideration and will be further evaluated within the COMET-trial.

## Supporting information

S1 File Semi-structured interview guide. (DOCX)

S2 File Codebook. (DOCX)
