# Supplementary material for: Psychotherapists’ perspectives on collaboration and stepped care in outpatient psychotherapy—A qualitative study
Source: PLoS One. 2020 Feb 5;15(2):e0228748. doi: 10.1371/journal.pone.0228748 (PMC7002019; doi:10.1371/journal.pone.0228748)
Supplement: S1 File — (DOCX) [file pone.0228748.s001.docx]

# **S1 File**

# **Semi-structured interview guide**

English version

## Collaboration

- Who are your most important collaboration partners?
- What would you wish for with regard to these collaboration partners?
- How do you experience the collaboration with general practitioners?
- How do you experience the collaboration with psychiatrists?
- How do you experience the collaboration with inpatient clinics?
- What do you think how your collaboration partners would evaluate the collaboration?

## Stepped Care

- What do you think of when thinking about “stepped care?

🡪 short input on stepped care if necessary

- What do you think about the concept of stepped care?
- What do you think about stepped care with regard to collaboration?

## Closure

- Are there any further aspects that you consider to be important and that we did not touch upon or too shortly?

German version

## Kooperation

- Wer sind für Sie als (ärztlicher) PsychotherapeutIn wichtige Kooperationspartner?
- Was würden Sie sich im Hinblick auf diese Kooperationspartner wünschen?
- Wie erleben Sie die Kooperation mit Hausärztinnen und Hausärzten?
- Wie erleben Sie die Kooperation mit Psychiaterinnen und Psychiatern?
- Wie erleben Sie die Kooperation mit stationären Einrichtungen?
- Was glauben Sie, wie Ihre Kooperationspartner die Zusammenarbeit beurteilen würden?

## Stepped Care

- Was fällt Ihnen zum Begriff „Stepped Care“ ein?

🡪 ggf. Input zum Stepped-Care-Konzept

- Was halten Sie vom Konzept der „Stepped Care“-Behandlung?
- Was denken Sie über Stepped Care in Bezug auf die Kooperation?

## Abschluss

- Gibt es noch weitere Aspekte, die Ihnen wichtig sind und die nicht oder zu kurz zur Sprache gekommen sind?
