# Supplementary material for: Psychotherapists’ perspectives on collaboration and stepped care in outpatient psychotherapy—A qualitative study
Source: PLoS One. 2020 Feb 5;15(2):e0228748. doi: 10.1371/journal.pone.0228748 (PMC7002019; doi:10.1371/journal.pone.0228748)
Supplement: S2 File — (DOCX) [file pone.0228748.s002.docx]

## **S2 File**

## **Codebook**

| 1 Stepped Care |
| --- |
| 1.1 definition |
| 1.1.1 stepped care - input |
| 1.1.2 term unknown |
| 1.1.3 proper description |
| 1.1.4 rather vague comprehension |
| 1.2 psychotherapists‘ perspective |
| 1.2.1 psychotherapists contribute to deficient health care offer |
| 1.2.2 „stepped“ care even within psychotherapy |
| 1.2.3 needs |
| 1.2.3.1 knowledge needed on treatment offers and providers |
| 1.2.3.1.1 "network coordinator" |
| 1.2.3.2 transparency needed regarding conditions and processes |
| 1.2.3.2.1 participation of private practices? |
| 1.2.3.2.2 participation of health insurances |
| 1.2.4 regulations on consultation hours 🡪 similar to stepping in stepped care |
| 1.3 skepticism/negative evaluation |
| 1.3.1 regarding novelty of the stepped care concept |
| 1.3.1.1 „already established“/“intuitive“ |
| 1.3.1.2 "Don’t we do this already?" |
| 1.3.2 regarding systematic monitoring |
| 1.3.2.1 questionnaires |
| 1.3.2.1.1 skepticism if therapy success is traceable while in therapy |
| 1.3.2.1.2 „red tape“ |
| 1.3.2.1.3 skepticism if therapy success is measurable with questionnaires |
| 1.3.3 "have handed you quite a bag of skepticism" |
| 1.3.4 regarding patients‘ perspective |
| 1.3.4.1 free choice of physicians and treatment should be preserved |
| 1.3.4.2 excessive demands on patients |
| 1.3.4.3 changing contact persons depending on treatment step 🡪burden for patients |
| 1.3.4.4 not all patients have a GP, esp. young patients |
| 1.3.4.5 „nips patients‘ responsibility in the bud“ |
| 1.3.5 regarding „economization“ of health care |
| 1.3.5.1 pre-determined therapy contingent creates demands |
| 1.3.5.2 presumption that questionnaire are introduced due to economic reasons |
| 1.3.5.3 „feeding off“ of patients with online treatment 🡪 delay of real treatment |
| 1.3.5.4 impression that treatment generally has to be faster or shorter |
| 1.3.5.5 regulations only due to economic reasons |
| 1.3.6 regarding own psychotherapeutic role |
| 1.3.6.1 endangering own „psychic hygiene“ |
| 1.3.6.2 Does „classical“ psychotherapy still have a place? |
| 1.3.6.3 relation to physicians |
| 1.3.6.3.1 only limited treatment options as psychotherapists 🡪 stepping not possible |
| 1.3.6.3.2 psychological psychotherapists are „bossed around“ by physicians |
| 1.3.6.3.3 greater competence than GPS, not adequately taken into consideration |
| 1.3.6.3.4 „territorial claims“ |
| 1.3.6.4 fear to lose treatment autonomy |
| 1.3.7 regarding content of stepped care |
| 1.3.7.1 skepticism |
| 1.3.7.1.1 treatment process should evolve „organically“ |
| 1.3.7.1.2 "Wo drafts the steps? Based on which evidence?" |
| 1.3.7.2 deficient diagnostic system 🡪 implementation in stepped care as well deficient |
| 1.3.8 regarding health care system |
| 1.3.8.1 free choice of physicians/ no steering unit fosters doctor hopping |
| 1.3.8.2 ambivalent physician’s role with trust/openness vs. provision of sick certificate |
| 1.3.8.3 lack of esteem of psychotherapists within health care system |
| 1.3.8.4 resentment with regard to consultation hours regulations |
| 1.3.8.5 lack of support by the „Kassenärztlichen Vereinigung“ |
| 1.3.8.5.1 "flagellation" |
| 1.3.9 regarding resources |
| 1.3.9.1 lack of time vs. time needed for stepped care |
| 1.3.9.1.1 esp. among GPs |
| 1.3.9.2 mutual availability |
| 1.3.9.3 lack of resources |
| 1.3.9.3.1 lack of psychotherapists 🡪 flawed demand planning |
| 1.3.9.3.2 resources for coordination are missing |
| 1.3.10 regarding competence |
| 1.3.10.1 lack of medical knowledge among psychological psychotherapists |
| 1.3.10.2 lack of knowledge on differential diagnostics and classification among GPs |
| 1.3.10.2.1 competences and interests very heterogeneous |
| 1.3.11 regarding collaboration |
| 1.3.11.1 skepticism if more collaboration is realistic and feasible |
| 1.3.11.2 has to be fostered by law |
| 1.3.11.3 previous negative experiences within „psychenet“ |
| 1.3.11.4 Who steers collaboration? worries about hierarchy |
| 1.3.11.5 needs time and space |
| 1.3.11.6 „simple“ collaboration between GPs would be enough |
| 1.3.11.7 collaboration can only work regionally |
| 1.3.11.8 requires efforts on all sides |
| 1.3.11.9 care providers cling to their autonomy |
| 1.3.12 regarding remuneration |
| 1.3.12.1 collaboration has to be rewarded financially |
| 1.3.12.2 lack of remuneration for required time/differential diagnostic/talking |
| 1.3.13 regarding efforts needed/“red tape“/control/coordination |
| 1.3.13.1 requires overview on treatment offers and providers |
| 1.3.13.2 fear of too much regulations |
| 1.3.13.3 would have to be enforced by law |
| 1.3.13.4 simple stepped care (usefulness?) vs. complex stepped care (too time consuming?) |
| 1.3.13.5 „red tape“ |
| 1.3.13.6 implementation 🡪who controls? |
| 1.4 positive evaluation |
| 1.4.1 regarding consultations hours for differential diagnostic and classification in steps |
| 1.4.2 generally |
| 1.4.2.1 increases transparency |
| 1.4.3 regarding early treatment initiation |
| 1.4.3.1 reasonable for facilitated access to in-patient care |
| 1.4.3.2 good if it counteracts chronification or deterioration |
| 1.4.3.3 good for better identifying patients with mental disorders |
| 1.4.3.3.1 good for diversifying GPs‘ diagnoses and treatment ideas |
| 1.4.3.4 good for informing patients on treatment options |
| 1.4.3.5 good if GPs serve as low-threshold access |
| 1.4.3.6 good for initiating treatment early |
| 1.4.4 regarding systematic monitoring |
| 1.4.4.1 approval of systematically adapting treatment/stepping-up and -down |
| 1.4.4.2 systematic re-appraisal important, at present deficient |
| 1.4.5 regarding improvement of GPs‘ competences |
| 1.4.5.1 esp. relevant for GPs |
| 1.4.5.2 implementable instruments would be helpful |
| 1.4.6 regarding stepping of care |
| 1.4.6.1 basically support of „stepping“-idea |
| 1.4.6.2 clearer care pathways would be helpful |
| 1.4.7 regarding collaboration |
| 1.4.7.1 esp. necessary for patients when severely ill/multimorbid/in crisis |
| 1.4.7.2 would foster collaboration/exchange |
| 1.4.7.3 positive experiences from previous study “psychenet“ |
| 2 collaboration |
| 2.1 most important collaboration partners |
| 2.1.1 GPs/ internists working as GPs |
| 2.1.1.1 esp. for somatoform patients |
| 2.1.2 specialists |
| 2.1.2.1 neurologists |
| 2.1.2.2 psychiatrists |
| 2.1.3 in-patient clinics/day-care clinics |
| 2.1.4 policlinics |
| 2.1.5 social psychiatric services |
| 2.1.6 helpdesks |
| 2.1.7 organizational infrastructure (reception, online platforms etc.) |
| 2.1.8 single mentions |
| 2.1.8.1 pain therapists |
| 2.1.8.2 co-care providers (e.g. in group therapy) |
| 2.1.8.3 occupational therapy |
| 2.1.8.4 custodian |
| 2.1.8.5 self-help groups/self-help coordination office |
| 2.2 collaboration intensity and form |
| 2.2.1 existing offers for exchange |
| 2.2.1.1 at times on events of the „Kassenärztlichen Vereinigung” |
| 2.2.2 regional networks |
| 2.2.3 developable |
| 2.2.4 from time to time |
| 2.2.5 not much interaction/rarely |
| 2.3 collaboration in somatoform disorders esp. important |
| 2.4 division of tasks can make sense (medical/psychotherapeutic) |
| 2.4.1 because of professional roles/psychodynamics |
| 2.4.2 division necessary due to treatment capacities/frequency |
| 2.5 general challenges/problems |
| 2.5.1 health care conditions |
| 2.5.1.1 lack of capacities/resources |
| 2.5.1.1.1 Does the effort pay off for own limited need for collaboration? |
| 2.5.1.1.2 no remuneration/lack of remuneration for other tasks |
| 2.5.1.1.2.1 e.g. lack of remuneration for medical statement for psychotherapy reimbursement |
| 2.5.1.1.3 lack of time |
| 2.5.1.1.3.1 for exchange |
| 2.5.1.1.4 mutual availability |
| 2.5.1.1.5 physicians do not want even more duties |
| 2.5.1.1.6 overcrowding of psychotherapists |
| 2.5.1.1.7 overcrowding of psychiatrists |
| 2.5.2 networking |
| 2.5.2.1 personal contacts/getting to know each other is essential |
| 2.5.2.2 networking in big cities more challenging |
| 2.5.2.3 access to existing networks challenging |
| 2.5.3 patient influence/ perspective |
| 2.5.3.1 collaboration as well dependent on patient’s will |
| 2.5.3.2 free choice of physicians |
| 2.5.3.2.1 patients influence collaboration and exchange due to choice of physicians |
| 2.5.3.3 lack of clarity regarding responsibilities in treatment (e.g. medication) |
| 2.5.4 delineation of different professional groups |
| 2.5.4.1 opinions on other specialties |
| 2.5.4.2 „territorial claims“ |
| 2.5.4.3 position of the psychotherapists |
| 2.5.4.3.1 lack of esteem for psychotherapists |
| 2.5.4.3.2 little knowledge on psychotherapeutic work and approaches |
| 2.5.4.4 clinging to „models and „codes“ of specific professional groups |
| 2.5.4.5 different/specialty-specific influence of physicians on patients |
| 2.5.5 acting under uncertainty |
| 2.5.5.1 there is no absolute diagnostic clarity |
| 2.5.5.2 self-critical attitude necessary |
| 2.6 differing definitions of „good collaboration“ |
| 2.7 reasons to collaborate |
| 2.7.1 transitions between care providers/transfer of care to other care providers |
| 2.7.1.1 "outsourcing of medication" due to psychodynamic reasons |
| 2.7.1.2 physicians are glad if they get rid of patients quickly |
| 2.7.1.3 questions regarding the discharge letter of clinics |
| 2.7.1.4 at the end of a psychotherapy |
| 2.7.1.5 if psychotherapy fails due to incompatibility between patient and therapist |
| 2.7.1.6 physicians‘ side: when there is no somatic reason but relevant psychological aspects |
| 2.7.2 for somatic diagnostics/care |
| 2.7.2.1 e.g. for exposure therapy |
| 2.7.2.2 for somatic care |
| 2.7.3 in complex cases |
| 2.7.3.1 if more intensive treatment is needed |
| 2.7.3.2 "when it gets complicated" |
| 2.7.3.3 in case of danger |
| 2.7.3.4 in case of „standstill“ in psychotherapy |
| 2.7.4 formal constraints |
| 2.7.4.1 somatic report for the reimbursement of psychotherapy |
| 2.7.4.2 sick certificate |
| 2.7.4.3 vocational reintegration |
| 2.7.5 only if needed |
| 2.7.5.1 when psychotherapists takes initiative |
| 2.7.5.2 only when patient consents |
| 2.7.5.3 if requested |
| 2.8 change of perspective (What do collaboration partners think about psychotherapists) |
| 2.8.1 no feedback 🡪 change of perspective difficult |
| 2.8.2 aspects that influence the assumed evaluation |
| 2.8.2.1 lack of time as major barrier |
| 2.8.2.1.1 overload on both sides 🡪 no exchange |
| 2.8.3 assumed negative or neutral evaluation |
| 2.8.3.1 "let them do their thing” |
| 2.8.3.2 partly collaboration partners with limited interest in psychotherapy |
| 2.8.3.3 negative opinion of some professional groups about psychotherapy |
| 2.8.3.3.1 orthopedists |
| 2.8.3.4 deficient availability of psychotherapists |
| 2.8.3.5 evaluation probably as „poor“ |
| 2.8.3.6 not interested |
| 2.8.3.7 need for more options to refer patients to/frustration about lack of options |
| 2.8.3.8 "reticent psychotherapists" |
| 2.8.3.9 "they laugh about us" |
| 2.8.4 assumed positive evaluation |
| 2.8.4.1 in principle wish for more collaboration |
| 2.8.4.2 interested |
| 2.8.4.2.1 esp. GPs because of excessive demands on their care provision |
| 2.8.4.3 "It works well." |
| 2.8.4.4 good in case of personal contacts /networks |
| 2.9 wishes for collaboration |
| 2.9.1 no wishes |
| 2.9.2 more collaboration for… |
| 2.9.2.1 ... better agreement, less parallel treatment („two tracks”) |
| 2.9.2.2 ... differential diagnostics in mental disorders |
| 2.9.3 more collaboration with ... |
| 2.9.3.1 ... physicians |
| 2.9.3.1.1 sometimes influence/intervention desirable |
| 2.9.3.1.2 "low-threshold networking" |
| 2.9.3.1.3 no influence on e.g. medication despite of intensive work within psychotherapy |
| 2.9.3.1.4 information gained in psychotherapy could be useful for physicians |
| 2.9.3.2 ... social services |
| 2.9.3.3 ... in-patient clinics |
| 2.9.4 ways to get into contact |
| 2.9.4.1 interdisciplinary meetings/ „quality circles“/intervision |
| 2.9.4.1.1 training for GPs |
| 2.9.4.1.2 case conferences |
| 2.9.5 conditions |
| 2.9.5.1 esp. need for timely appointments |
| 2.9.5.2 more time on all sides |
| 2.9.5.2.1 more personal contacts (but: lack of time) |
| 2.9.5.3 improved availability |
| 2.9.5.3.1 esp. regarding GPs |
| 2.9.5.3.2 special numbers for contact/special consultation hours |
| 2.9.5.4 remuneration for collaboration |
| 2.9.5.5 change on the health care system level |
| 2.9.6 mutual esteem |
| 2.10 ... with in-patient clinics/ day-clinics |
| 2.10.1 aspects that influence collaboration |
| 2.10.1.1 lack of time/mutual availability |
| 2.10.2 reasons for collaboration |
| 2.10.2.1 for referral |
| 2.10.3 quality/intensity/ways of collaboration |
| 2.10.3.1 heterogeneous |
| 2.10.3.2 "good" |
| 2.10.3.2.1 single good contacts |
| 2.10.3.2.1.1 personal contacts extremely helpful |
| 2.10.3.3 "from time to time" |
| 2.10.3.4 no direct/real collaboration |
| 2.10.3.5 discharge letter = sufficient |
| 2.10.3.6 contact rather via patients |
| 2.10.4 criticism |
| 2.10.4.1 direct contact difficult |
| 2.10.4.2 waiting-lists too long, wish for facilitated access |
| 2.10.4.2.1 differences in the possibility to influence waiting-lists |
| 2.10.4.2.2 "really frustrating!" |
| 2.10.4.3 long waiting time for discharge letter |
| 2.10.4.4 criticism regarding additional diagnoses given by clinics |
| 2.10.4.5 "crude" assumptions of some clinics regarding outpatient psychotherapy |
| 2.11 ... with physicians in general |
| 2.11.1 negative |
| 2.11.1.1 rarely/only when needed |
| 2.11.1.2 unpleasing contact |
| 2.11.1.3 no interest on the physicians‘ side |
| 2.11.2 positive |
| 2.11.2.1 if there is contact 🡪 helpful |
| 2.11.2.2 contact maybe depends on shared medical background |
| 2.12 ... with helpdesks |
| 2.13 … with other medical specialties |
| 2.13.1 clinging to specific professional „models“ |
| 2.14 ... with psychiatrists |
| 2.14.1 aspects that influence collaboration |
| 2.14.1.1 difficult mutual availability, lack of time |
| 2.14.1.2 generally lack of psychiatrists |
| 2.14.2 negative |
| 2.14.2.1 partly „colliding models“ |
| 2.14.2.2 no equal rights |
| 2.14.3 reasons to collaborate |
| 2.14.3.1 medication |
| 2.14.4 positive |
| 2.14.4.1 single good contacts/regional networks |
| 2.14.4.1.1 trying to be available for each other |
| 2.14.4.1.2 personal contact/“same wavelength“ important |
| 2.14.4.2 in principle willing to collaborate |
| 2.14.4.3 good for getting a second opinion |
| 2.14.5 very heterogeneous |
| 2.14.6 rarely |
| 2.15 ... with GPs/internists working as GPs |
| 2.15.1 aspects that influence collaboration |
| 2.15.1.1 direct exchange not considered necessary |
| 2.15.1.2 contact depending on own engagement |
| 2.15.1.3 lack of time/mutual availability |
| 2.15.1.4 personal contact essential |
| 2.15.2 very heterogeneous |
| 2.15.3 positive |
| 2.15.3.1 regional networks |
| 2.15.3.1.1 requires time, grows over time |
| 2.15.3.2 (rather) good |
| 2.15.3.2.1 single good contacts |
| 2.15.3.2.1.1 trying to be available for each other |
| 2.15.3.2.1.2 „on the same wavelength“ |
| 2.15.3.3 exchange of information important |
| 2.15.3.5 compared to other medical specialties relatively open for „psycho-models“ |
| 2.15.4 negative |
| 2.15.4.1 rarely direct contact |
| 2.15.4.1.1 contact easier than with psychiatrists |
| 2.15.4.2 "sometimes very strenuous" |
| 2.15.4.3 no interest on the GPs‘ side |
| 2.15.4.3.1 "tired of the system" |
| 2.15.4.3.2 not trained well enough for psychiatric medication |
| 2.15.4.3.3 wanting to refer patients to psychiatrists, but lack of psychiatric care |
| 2.15.4.4 no equal status, GPs don’t take psychotherapists seriously |
| 2.15.4.5 criticism regarding limited psychological competence of GPs |
| 2.15.4.6.1 too many sick certificates |
| 2.15.4.6.2 flawed referrals |
| 2.15.4.6.3 too many psychiatric diagnoses/too quick in prescribing psychopharmacotherapy |
| 2.15.4.7 lack of esteem of GPs for psychotherapists |
| 2.15.5 reasons to collaborate/ways of getting into contact |
| 2.15.5.1 for reimbursement of psychotherapy |
| 2.15.5.2 exchange of information via patients |
| 2.15.5.3 rather in written form |
| 2.15.5.4 medication |
